# Rotating Stomata Measurement Based on Anchor-Free Object Detection and Stomata Conductance Calculation

**DOI:** 10.34133/plantphenomics.0106

**Published:** 2023-10-09

**Authors:** Fan Zhang, Bo Wang, Fuhao Lu, Xinhong Zhang

**Affiliations:** ^1^ Huaihe Hospital of Henan University, Kaifeng 475004, China.; ^2^ Henan Key Laboratory of Big Data Analysis and Processing, Henan University, Kaifeng 475004, China.; ^3^ State Key Laboratory of Crop Stress Adaptation and Improvement, Henan University, Kaifeng 475004, China.; ^4^ School of Software, Henan University, Kaifeng 475004, China.

## Abstract

Stomata play an essential role in regulating water and carbon dioxide levels in plant leaves, which is important for photosynthesis. Previous deep learning-based plant stomata detection methods are based on horizontal detection. The detection anchor boxes of deep learning model are horizontal, while the angle of stomata is randomized, so it is not possible to calculate stomata traits directly from the detection anchor boxes. Additional processing of image (e.g., rotating image) is required before detecting stomata and calculating stomata traits. This paper proposes a novel approach, named DeepRSD (deep learning-based rotating stomata detection), for detecting rotating stomata and calculating stomata basic traits at the same time. Simultaneously, the stomata conductance loss function is introduced in the DeepRSD model training, which improves the efficiency of stomata detection and conductance calculation. The experimental results demonstrate that the DeepRSD model reaches 94.3% recognition accuracy for stomata of maize leaf. The proposed method can help researchers conduct large-scale studies on stomata morphology, structure, and stomata conductance models.

## Introduction

Stomata play an essential role in facilitating gas and water exchange between terrestrial plant leaves and atmosphere. Plants have developed this unique mechanism for controlling gas and water exchange due to the transition from aquatic to land environments [1,2]. By regulating the degree of opening and closing, stomata control critical processes such as photosynthesis and gas transpiration, which ultimately influence plant metabolism [3]. Consequently, the study of stomata is integral to understanding of how plants regulate their ecological environment [4,5].

During plant growth, several factors can influence the features of stomata, including hormones, light conditions, and atmospheric CO _2_ concentration [6,7]. Stomata density and size tend to increase with higher light intensity and decrease with increasing CO _2_ concentration [8–11]. Stomata gas exchange capacity is determined by various factors, such as density, size, and pore size [12–14]. The opening and closing of stomata are regulated by the variation of pore aperture because the pore length is relatively fixed [15,16]. These stomata traits have been extensively studied and are often used to estimate stomata conductance, which is an indicator of the degree of stomata opening and closing [17–19]. Stomata conductance is inversely proportional to stomata resistance and provides insight into the plant’s gas exchange with gases such as carbon dioxide and water vapor [20].

Traditional methods of measuring stomata typically involve using optical microscopy to manually observe and measure them. However, these methods often require researchers to manually mark the stomata’s features, such as boundary, length, and width, which are time-consuming and prone to human error. To address these issues and improve efficiency, researchers have developed automated measurement methods for stomata detection.

Omasa and Onoe [21] initially proposed a method to measure stomata anatomical parameters, which employed Fourier transform and un-sharp masking technique to eliminate noises from the original images. Their method measured the length and width of sunflower stomata by border detection. However, their method has some limitations, such as its computational complexity and its applicability to single porosity images only. To address this issue, Laga et al. [22] developed an automated method using template matching to detect stomata and binary segmentation to extract stomata aperture. Nonetheless, this automated method relied on templates for each plant species. To overcome this drawback, Liu et al. [23] employed optimal stable external regions [metasurface-enhanced Raman spectroscopy (MSER)] for grapevine stomata detection and measurement. It was a semi-automatic approach, as it required the user to correctly choose the ellipse to accommodate different stomata. In contrast, Jayakody et al. [24] introduced a fully automatic method for measuring stomata in grape varieties, based on machine learning theory. This method built a cascaded object detector for detecting stomata using histogram of the oriented gradient (HOG) feature, and further calculated various relevant parameters using binary-image segmentation and skeleton techniques. However, this method requires that the microscopic image of stomata contains affluent background features. Although these methods outperform purely manual methods when measuring stomata traits, some limitations still exist.

Due to the advancement of deep learning (DL) techniques, it has become achievable to efficiently and accurately identify and measure stomata. Researchers have proposed numerous methods for stomata identification utilizing DL techniques. Bhugra et al. developed a DCNN (deep convolution neural network)-based model for stomata detection [25]. Sakoda et al. evaluated the density of stomata in soybean leaf and examined the variation utilizing a high-throughput technique [26]. Fetter et al. presented a DCNN-based stomata automatic counting system that achieved high accuracy in identifying stomata in various microscopic images [27]. To obtain the coordinates of stomata contour, Song et al. [28] proposed a DCNN-based stomata automatic segmentation and detection method using the Mask R-CNN model. Additionally, Casado-Garcia et al. developed a stomata detection method, named LabelStomata, for various plant leaves [29], and Meeus et al. demonstrated the applicability of a deep neural network-based stomata automatic detection method in angiosperm phylogeny using a leaf-to-label workflow [30]. Millstead et al. realized automatic detection of stomata by using CNN and proposed a novel binary-image segmentation approach and cross-sectional analysis approach to obtain stomata boundaries and associated regions. [31].

Despite the marked advancements in DL-based methods, they often disregard the unique features of plant stomata images and fail to address challenges in accelerating convergence speed and improving model generalization. Although some DL-based methods can automatically identify and calculate stomata, they fall short in measuring stomata parameters simultaneously and require manual processing at a later stage. Moreover, these methods do not integrate DL with stomata conductance analysis. The traditional analysis methods of stomata traits are either mainly non-automatic observation and measurement, or semi-automatic analysis techniques, which are inefficient, labor-intensive, and difficult to automate. Although some automatic stomata identification and counting methods have been proposed, they are based on horizontal anchor boxes and cannot identify the rotating stomata well, which affects the efficiency of stomata trait analysis.

In this paper, we propose a novel anchor-free approach, named DeepRSD (deep learning-based rotating stomata detection), for detection and measurement of rotating stomata at the same time. Our approach can automatically identify stomata in maize leaves, measure associated traits, and calculate stomata conductance.

The main contributions of this paper are as follows:

a. A DeepRSD method is proposed based on anchor-free object detection networks.

b. Stomata detection and measurement can be done simultaneously, without the need for other processing (such as rotating images).

c. Adding an angle detection head to DeepRSD model to improve the selection accuracy of rotating stomata detection.

d. Adding a stomata conductance loss function to DeepRSD model training to measure the stomata traits and the stomata conductance more accurately.

This paper is organized as follows: The “Introduction” section deals with the introduction. The “Materials and Methods” section introduces the materials and methods used in this research. The “Results” section presents the experimental results. The “Discussion” section provides a discussion. Finally, conclusions are drawn in the “Conclusions” section.

## Materials and Methods

**Fig. 1.**
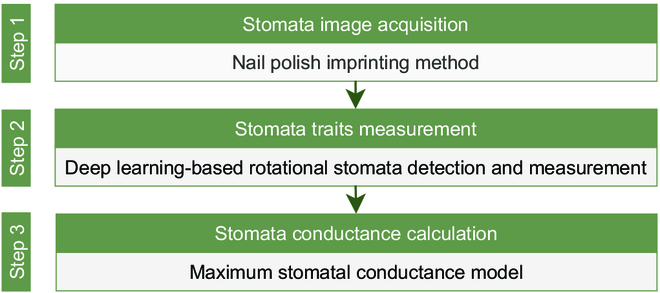
Schematic diagram of the steps for automatic detection of rotating stomata and calculation of stomata conductance.

The proposed rotating stomata detection and measurement approach is divided into 3 steps. First, microscopic images of stomata are obtained through the nail polish imprinting method. Second, DeepRSD model is used to detect stomata in plant leaf images and 3 stomata traits are extracted, namely, stomata length, lumen depth, and density. Last, the maximum stomata conductance model algorithm is used in conjunction with the stomata traits extracted in step 2 to calculate stomata conductance. Figure 1 shows the schematic diagram of steps for automatic detection of rotating stomata and calculation of stomata conductance.

### Image acquisition

The maize germplasm was obtained from the Centro Internacional de Mejoramientode Maizy Trigo (CIMMYT) [32]. The maize samples were cultivated in Yuan Yang County, Henan Province. The longitude and latitude of the experimental site are 113.947402°E and 35.112807°N, respectively.

The nail polish imprinting approach was used to obtain stomata image of the upper epidermis of maize leaves. At a temperature of 25 ^∘^C, transparent nail polish was evenly applied to the middle of the maize leaf, waiting 20 min for the transparent nail polish to dry. Further, the dried nail polish layer was removed with tweezers. A 1.5-ml test tube is used for collection of the nail polish layer for storage. The nail polish layer was carefully removed from maize leaf with tweezers and placed on a slide. To make the nail polish layer fit the slide, we add a small amount of water and then add the lid. Then, we observed the slide using optical microscope at magnification of 10×10 and 10×20. The final stomata image added to the dataset was randomly captured from a clear field of view using ImageView software. Finally, a total of 2,192 maize leaf images were obtained for the next experimental analysis.

### Stomata image preprocessing

Preprocessing of original microscopic stomata images is necessary during the experiments. In this paper, preprocessing refers to data augmentation. In DL, data augmentation refers to the method of increasing the amount of data by adding small changes to existing data or creating new synthetic data from existing data. We collected a total of 2,192 stomatal images. Such amount of data is not enough for DL model training, so we need to enhance the data. Data augmentation can increase the diversity of data samples and improve the robustness of the model at the same time to reduce the risk of overfitting. The data enhancement methods we used include geometry correction algorithm and grayscale stretching algorithm. In the geometric correction algorithm, we enhance the data by rotating the image; specifically, we rotate the image by 30°, 45^°^, and 90^°^. Grayscale stretching is a method of changing the contrast of an image. Through grayscale mapping, grayscale values in one section of the original image are mapped to another grayscale value, thus stretching or compressing the entire range of grayscale distribution of the image. We set the grayscale value of the original stomata image between 210 and 255, which makes the stomata in the image more contrasting with the background. The utilization of geometry distortion correction and gray-scale stretching algorithms can effectively amplify the contrast between stomata and background, thus improving recognition and segmentation performance.

**Fig. 2.**
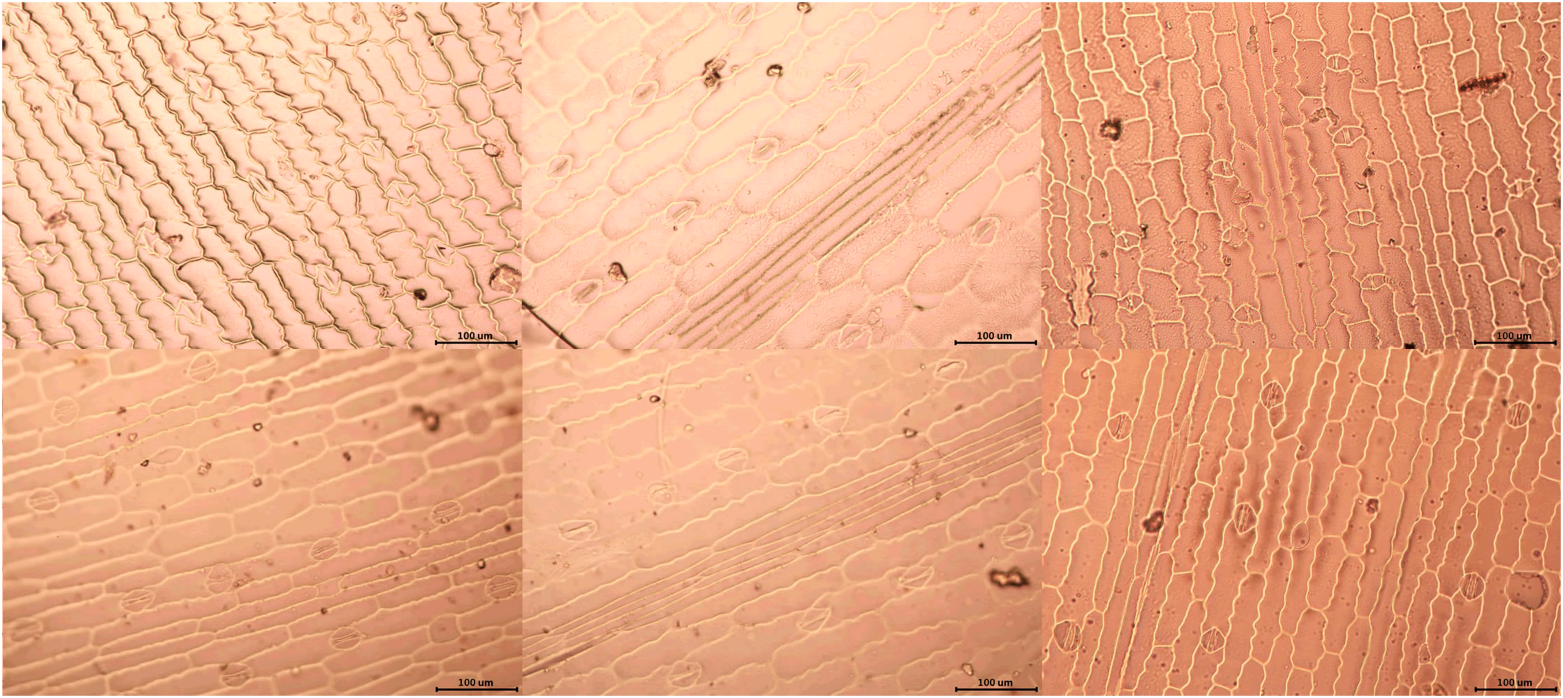
Stomata images of maize leaves after preprocessing.

DL-based object detection methods require extensive training on a representative dataset. During training, the DL model computes the loss between the predicted values and true values by analyzing labeled images. Subsequently, it adjusts and optimizes the model’s parameters to minimize the errors and improve its performance. The quality of the training dataset significantly affects the model’s accuracy. Larger datasets generally produce better results. Furthermore, certain sample images must be manually labeled for DL training. Figure 2 displays preprocessed maize leaf images obtained through the nail polish imprinting method.

### Anchor-free object detection

The anchor-based object detection algorithm needs to calculate all the anchor networks on the image and then classify the anchor networks into background and object, which is a relatively time-consuming and inefficient method. The anchor-free network-based algorithm proposes a new way of thinking, and the position of the object is locked by calculating the key point position of the object and later regressing the size of the object by the width and height feature information.

As an end-to-end model, CenterNet is faster and more accurate in inference than anchor network-based models [33]. The CenterNet model first generates a key point heatmap for the input image and then scales to a preset size. The locations of the key points in the original image are then down-sampled and dispersed into the heatmap by Gaussian distribution, accounting for errors due to data dispersion. The centroids of all objects are obtained by key point estimation.

The peak point of each category is extracted from the heatmap, and the value points in the 8 adjacent regions that are greater than or equal to that point are also detected. The information of the first 100 peak points is saved, and the key point value is used as a metric of detection confidence. The size of the object is calculated based on centroid regression. All outputs can be generated directly from key point estimation without the need for intersection over union (IoU)-based nonmaxima suppression (NMS) or other subsequent processing. The NMS method is replaced by extracting the peak key points of the heatmap using a 3×3 max pooling operation.

The backbone structure in our approach uses the DLA (deep layer aggregation) network, which first extracts the feature map using the DLA-based module and continuously shrinks the feature map to learn the higher-level semantic features. The DLA structure consists of 2 parts: iterative deep aggregation (IDA) and hierarchical deep aggregation (HDA) [34].

#### Iterative deep aggregation

**Fig. 3.**
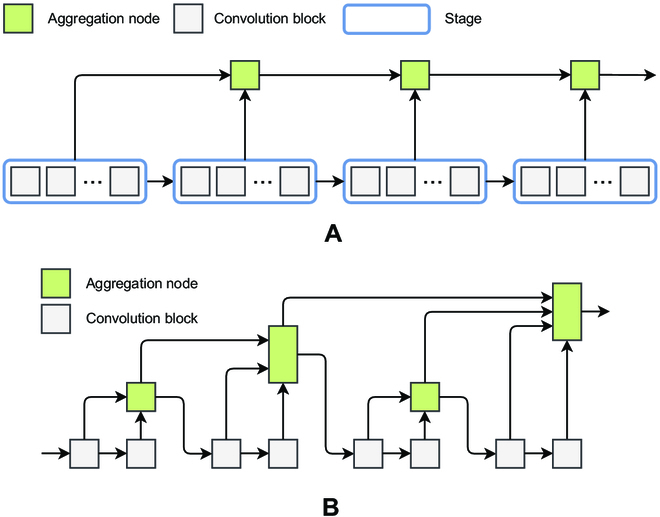
(A) IDA module structure diagram. (B) HDA module structure diagram.

As the network structure deepens, the semantic information in the network hierarchy becomes stronger, but the spatial information becomes coarser. The IDA module aggregates features from the shallowest and smallest scales and iteratively aggregates features at deeper scales and larger scales. In this way, shallower network information can also be processed in subsequent stages, resulting in a network structure that better captures the features of the input image. The IDA module is shown in Fig. 3A.

In Fig. 3A, the aggregation nodes aggregate features, while the features propagate from shallow to deep layers. In this case, the CNN structure is split. A CNN consists of multiple stages with consistent resolution between stages. The stages consist of multiple blocks, each containing multiple layers.

The formula for calculating the IDA process is shown in Eq. 1.

$$I\left(X_{1},\ldots ,X_{n}\right)=\left\{\begin{array}{cc} X_{1}, & \text{if}\;n=1\\ I\left(N\left(X_{1},X_{2}\right),\ldots ,X_{n}\right), & \text{otherwise}, \end{array}\right.$$ (1)

where *I* denotes the whole IDA module and *N* denotes an aggregation node. For example, *N*(*X*_1_, *X*_2_) denotes an aggregation node with inputs *X*_1_ and *X*_2_ .

#### Hierarchical deep aggregation

IDA can effectively fuse features from multiple stages; however, the features from multiple blocks within a stage cannot be fused. The HDA structure is used to enhance the fusion of multiple blocks within a stage. The features in the aggregated nodes are introduced into the backbone network through different down-sampling rates so that the current block takes the features from the previous aggregation as input. Through the HDA structure, shallow and deep network layers can be combined so that the combined information can span across the layers and thus the learned features are richer and more diverse. The HDA module is shown in Fig. 3B.

The HDA process is calculated as shown in Eq. 2.

$$T_{n}\left(x\right)=N\left(R_{n-1}^{n}\left(x\right),R_{n-2}^{n}\left(x\right),\ldots ,R_{1}^{n}\left(x\right),L_{1}^{n}\left(x\right)L_{2}^{n}\left(x\right)\right),$$ (2)

where *N* represents the aggregation node. The definitions of *R* and *L* are shown in Eqs. 3 to 5.

$$L_{2}^{n}\left(x\right)=B\left(L_{1}^{n}\left(x\right)\right),$$ (3)

$$L_{1}^{n}\left(x\right)=B\left(R_{1}^{n}\left(x\right)\right),$$ (4)

$$R_{m}^{n}\left(x\right)=\left\{\begin{array}{cc} T_{m}\left(x\right), & \text{if}\;m=n-1\\ T_{m}\left(R_{m+1}^{n}\left(x\right)\right), & \text{otherwise}, \end{array}\right.$$ (5)

where *B* represents the convolution block.

Following the above introduction, it is clear that the aggregation node has 2 inputs in the IDA structure, while in the HDA structure, the aggregation node has 2 or more inputs. The aggregation node fuses multiple input features to form a single feature and outputs it. To reduce the computational effort, a “convolution-BN-activation” function structure is used to construct the aggregation nodes with residual connections, as shown in Eq. 6.

$$N\left(x_{1},x_{2},\ldots ,x_{n}\right)=\sigma \left(\text{BatchNorm}\left(\sum_{i}‍W_{i}x_{i}+b\right)+x_{n}\right),$$ (6)

where *W_i_* denotes the weight and *b* denotes the bias.

#### DLA module structure

**Fig. 4.**
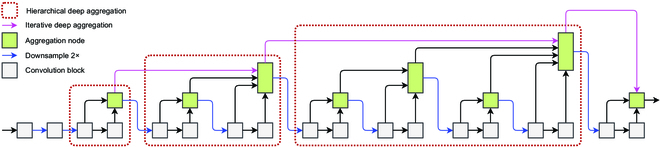
DLA model structure diagram.

As shown in Fig. 4, the IDA and HDA structures are combined to form the DLA structure. Each red dashed box in Fig. 4 can be considered as a stage. Multiple different stages are connected using IDA, and down-sampling operations are performed between each stage. The features within the stages are fused using HDA. IDA and HDA share the aggregation node.

### DeepRSD model

**Fig. 5.**
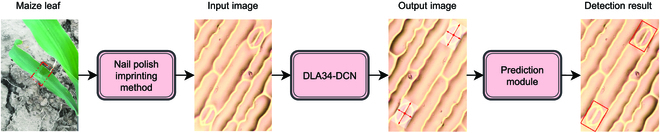
Flowchart of rotating stomata detection method for maize leaves.

We propose a DeepRSD model based on anchor-free networks. DeepRSD employs DLA-34 as the backbone network. Following the idea of CenterNet [33], deformable convolution networks (DCNs) [35], instead of traditional 2D convolution, are used to enhance the learning ability of deformed objects and obtain a larger perceptual field. An attention mechanism module is also introduced in the DeepRSD model, which pays more attention to the key information in the image, reduces the attention to other irrelevant information, and improves capability of the network to extract key features. The flowchart of rotating stomata detection method for maize leaves is shown in Fig. 5.

In the DLA network, the down-sampling layer usually uses either a maximum pooling or an average pooling operation. The output of the down-sampling layer is the feature map. The convolutional layers at the bottom of the DeepRSD use smaller step lengths so that more feature information can be retained, while the higher convolutional layers use larger step lengths to reduce the feature map size. This layered sampling method can enhance effectiveness and robustness. Meanwhile, the layer aggregation method in the DLA network can effectually resolve the gradient disappearance and gradient explosion problems in the network, thus improving the training efficiency and accuracy of the network.

Similar to CenterNet [33], we also use the DCN module in the up-sampling process. Traditional transposed convolutional operations usually use a fixed-shaped convolutional kernel to up-sample the feature map, which may cause some specific structures in the image (e.g., thin lines or small objects) to lose detailed information after up-sampling, thus affecting the accuracy of the model. DCN can adaptively adjust the shape of the convolution kernel according to the different positions of the input feature map, hence better preserving the information of specific structures and improving the up-sampling effect.

The principle of deformable convolution is to introduce a learnable offset matrix into the convolution operation and to achieve adaptive sampling operation of feature map by adjusting the sampling position of each position in the convolution kernel. Specifically, for each position of input feature maps, the deformable convolution calculates a new sampling position based on the offset and then performs the convolution operation centered on that position. The deformable convolution allows the convolution kernel to adaptively sample different regions of the input feature map. Thus, information such as object deformation and attitude changes can be better captured.

An attention module is introduced in the DLA-34 network to further improve the feature representation and enhance the network’s performance. After the last convolutional layer of the dense block, a CBAM (convolutional block attention module) module is used to weigh the output of this layer with attention to enhance the feature representation performance of the DLA-34 network. The main function of the attention mechanism is to adjust the weights of each position in feature maps adaptively according to the disparate feature contribution levels. Attention mechanism improves the network’s attention to important information and thus enhances the feature representation. Channel attention can learn channel weights and weight the features of different channels according to the interrelationships between them, while spatial attention can learn spatial weights and weight the features of different positions according to the interrelationships between them.

**Fig. 6.**
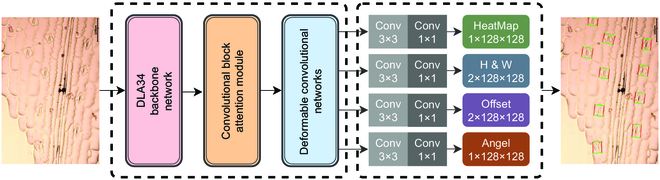
Structure of the DeepRSD model.

We added an angle detection head to the DeepRSD model. The angle detection head can further improve the accuracy of anchor-free object detection; thus, the enclosing box can better mark the stomata. In addition, we modified the loss function for DeepRSD model training so that the DeepRSD model can optimize multiple objects at the same time, such as centroid, size, and rotation angle. Figure 6 shows the structure of the DeepRSD model.

### Maximum stomata conductance model

Stomata traits have an important influence on plant carbon and water cycling processes. Therefore, accurate simulation of stomata behavior is key to predicting the effects of global change on vegetation structure and function. At present, the simulation of stomata conductance has progressed from empirical models to mechanistic models. The empirical models try to find statistical relationships between stomata conductance with environmental factors and plant physiological factors, while the mechanistic models have a better theoretical basis and can integrate a variety of environmental factors to obtain more biologically meaningful parameters.

In this paper,the stomata conductance calculation leverages the maximum stomata conductance algorithm [36]. The maximum stomata conductance model has been widely used to measure the effects of various factors on stomata traits, such as nitrogen, phosphorus, and potassium. The effects of different factors on stomata can be well reflected in plant stomata traits by maintaining the temperature at 25 ^∘^C. To better help the researchers concerned, this paper combines the maximum stomata conductance model to calculate the stomata conductance values of maize leaves.

In the DeepRSD model, we introduce an angle detection module to better detect the orientation and shape of the object. In particular, when identifying stomata, we can use the angle information of stomata to adjust the orientation and shape of the enclosing box to fit the stomata intact, thus improving the accuracy of stomata identification. Using this method, we can directly calculate the number of stomata as well as basic traits such as length and width, and then the stomata density is calculated according to the actual size and scale of stomata image. Finally, the maximum stomata conductance model is adopted to calculate stomata conductance of maize leaves. The advantage of this method is that it enables an end-to-end calculation of stomata conductance without additional intermediate processing. In addition, our method has good generality so it can be applied to other similar object detection and computation problems.

The maximum stomata conductance model is shown in Eq. 7.

$$G_{\text{smax}}=\frac{d\times \mathrm{SD}\times \alpha_{\text{max}}}{1.6v\left(l+\frac{\pi }{2}\sqrt{\frac{\alpha_{\text{max}}}{\pi }}\right)},$$ (7)

where *d* is the diffusivity of water in the air, *SD* is the stomata density (number of stomata per square millimeter), *v* is the molar volume of air, and *l* is the stomata cavity depth. It is usually assumed that stomata cavity depth is equal to the width of the guard cells. *α*_max_ is the estimated maximum stomata aperture area.

$$\alpha_{\text{max}}=\pi \times SL^{2}/4,$$ (8)

where *SL* is the stomata length.

In the experiments of this paper, we calculate the maximum stomata conductance in the ideal state. We set *d* = 24.9 × 10^−6^ m^2^ s^−1^ and *v* = 22.4 × 10^−3^ m^3^ mol^−1^ at 25 ^∘^C and 101.3 kPa.

### Loss function of DeepRSD training

The difference in the heatmap, width, height, and offset values between the predicted anchor boxes and the ground truth is calculated continuously in the DeepRSD model training. This difference is the loss value. In this paper, we add an angular loss function and a stomata conductance loss function, which allows the model to measure the stomata traits and conductance more accurately. Focal loss is adopted for the calculation of the difference between the predicted heatmap information and the real heatmap information. The heatmap loss function is shown in Eq. 9.

$$L_{H}=\left\{\begin{array}{cc} -\frac{1}{N}\sum_{i=1}^{N}‍{\left(1-p_{i}\right)}^{\alpha }\text{log}\left(p_{i}\right), & \text{if}\;q_{i}=1\\ -\frac{1}{N}\sum_{i=1}^{N}‍{\left(1-q_{i}\right)}^{\beta }{\left(p_{i}\right)}^{\alpha }\text{log}\left(p_{i}\right), & \text{otherwise}, \end{array}\right.$$ (9)

where *p* and *q* denote the heatmap value of the image prediction and the heatmap value of the real image, respectively. *i* is the index of the pixel positions in the feature map, and *n* is the number of pixels. *α* and *β* are 2 hyperparameters used to control the contribution of each point. In this paper, we set *α* = 2 and *β* = 4 based on the experiences of [37].

To minimize the difference between the predicted and true values, the rest of the loss functions are optimized using the L1 loss function. The centroid offset value loss is shown in Eq. 10.

$$L_{\text{off}}=\frac{1}{N}\sum_{k}‍|{\hat{O}}_{k}-O_{k}|,$$ (10)

where ${\hat{O}}_{k}$ and *O_k_* denote the offset information of predicted and true *k*th instance, respectively.

The length and width loss is shown in Eq. 11.

$$L_{\text{size}}=\frac{1}{N}\sum_{k}‍|{\hat{S}}_{k}-S_{k}|,$$ (11)

where ${\hat{S}}_{k}$ and *S_k_* denote the width and height information of the predicted and true *k*th instance, respectively.

The angular loss is shown in Eq. 12.

$$L_{\text{ang}}=\frac{1}{N}\sum_{k}‍|{\hat{\theta }}_{k}-\theta_{k}|,$$ (12)

where ${\hat{\theta }}_{k}$ and *θ_k_* denote the angle information of the predicted and true *k*th instance, respectively.

The stomata conductance loss is shown in Eq. 13.

$$L_{\text{con}}=\frac{1}{N}\sum_{k}‍|{\hat{G}}_{k}-G_{k}|,$$ (13)

where ${\hat{G}}_{k}$ and *G_k_* denote the stomata conductance information of the predicted and real *k*th instance, respectively. The stomata conductance is calculated according to the maximum stomata conductance model (Eq. 7). This model has been described in the “Maximum stomata conductance model” section.

The above loss functions are summed to evaluate the total loss function, as shown in Eq. 14.

$$L=L_{H}+L_{\text{off}}+L_{\text{size}}+L_{\text{ang}}+L_{\text{con}}.$$ (14)

## Results

### Image preprocessing and stomata labeling

The preprocessing of stomata images involves geometric correction algorithms and grayscale stretching algorithms. To ensure consistency, the images are converted from tiff format to jpg format, and the resolution is identically set to 1,000×667 pixels. The actual size is about 0.625 μm/pixel. In addition, some stomata images require manual labeling for DL training. When the stomata are located at the image boundaries, it is not possible to mark the stomata with a whole rotated box. To solve this problem, we add a 30-pixel-wide black border around each image. Additionally, some stomata are only partially displayed in the images. To address this, we specify that if the portion of the stomata in the image accounted for more than two-thirds of the total size of the stomata, then it will be considered a valid object for labeling.

The reason for labeling is that the ground-truth box of an object needs to be provided in the training of the DL model. The parameters of DeepRSD model are adjusted continuously by calculating the loss between the predicted value and the true value, which makes the predicted result closer to the true value. Finally, the stomata can be identified by the DL model accurately.

Typically, the labeled data are acquired by manually labeling stomata on stomata images using rectangular boxes. But manual labeling of image datasets always requires the assistance of software. Commonly used labeling software include labelme and labelimg2. In this research, labelimg2 is utilized to label the stomata images. The smallest enclosing rectangle is adopted to fit the stomata as closely as possible in the labeling process. After labeling all the stomata, the stomata label information is normalized and the label file including the stomata coordinate is labelimg2 software automatically. Finally, labelimg2 converts the label file to a JSON format, which can be recognized by the DeepRSD DL model.

### DL training and validation

The GPU we used is GeForce RTX 3060. The experimental software environment is Pycharm 2021.2, Anaconda3, Python3.8, Pytorch-GPU version 1.11.0, and Windows10 (Cuda version 11.3, cudn version 8.0 for GPU acceleration).

In this research, we collect 2,192 maize leaf images. The number of stomata images is not sufficient for the training of the DL model, so we did data enhancement. Eventually, the number of stomata images reached 24,112, in which the effective stomata objects are up to more than 100,000, which can completely meet the training requirements of the DeepRSD model. The ratio of training set to validation is set to 3:1. The training set is leveraged to learn stomata features, identify stomata, and optimize network parameters. The validation set is leveraged to assess the stomata recognition performance of the DeepRSD model.

During the DeepRSD model training, AdamW optimization algorithm is adopted. Weight decay is set to 5 × 10^−3^. Learning rate is continuously adjusted as the training progresses. By adjusting the learning rate during training, we can control the convergence speed and stability of the optimization algorithm. The learning rate adjustment strategy we adopt is exponential decay. The learning rate is decayed as shown in Eq. 15.

$$\mathrm{lr}=\mathrm{lr}\times \text{gamm}a^{\text{epoch}},$$ (15)

where the initial value of *lr* is set to 1.25 × 10^−3^, gamma is set to 0.95, and epoch is set to 20.

During the training of a DL model for object detection, our goal is to optimize the parameters of the DL model to minimize the loss functions associated with these parameters. In the DeepRSD model, the anchor-free stomata detection problem is turned into an optimization problem by using 5 distinct loss functions during the training process (Eq. 14). The loss functions include heatmap loss function, width–height loss function, offset value loss function, angular loss function, and stomata conductance loss function. Each loss function is designed to address specific aspects of the object detection problem. These loss functions help our model learn the complex patterns and features of objects, and improve the model’s ability to accurately detect objects in the input image.

**Fig. 7.**
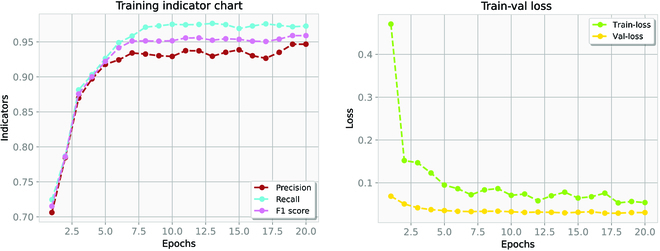
Parameter variation of the DeepRSD model during the training process. (A) Training indicator chart. (B) Train-val loss.

Figure 7 presents the parameter variation of the DeepRSD model during the training process. The training loss and validation loss plots indicate the changes in the loss function of the training set and the validation set. The Precision, Recall, and F1 score plots show the changes of the 3 parameters. Precision is the proportion of true examples among all positive predictions. Recall is the percentage of correct predictions among all positive cases (for example, the coverage of correct predictions). Based on the values of TP (true positive), FP (false positive), FN (false negative), and TN (true negative), the calculation formulae of Precision and Recall are shown in Eqs. 16 and 17.

$$\text{Precision}=\frac{\mathrm{TP}}{\mathrm{TP}+\mathrm{FP}},$$ (16)

$$\text{Recall}=\frac{\mathrm{TP}}{\mathrm{TP}+\mathrm{TN}}.$$ (17)

The calculation formula of F1 score is shown in Eq. 18.

$$F1\;\text{score}=2\times \frac{\text{Precision}\times \text{Recall}}{\text{Precision}+\text{Recall}}.$$ (18)

**Fig. 8.**
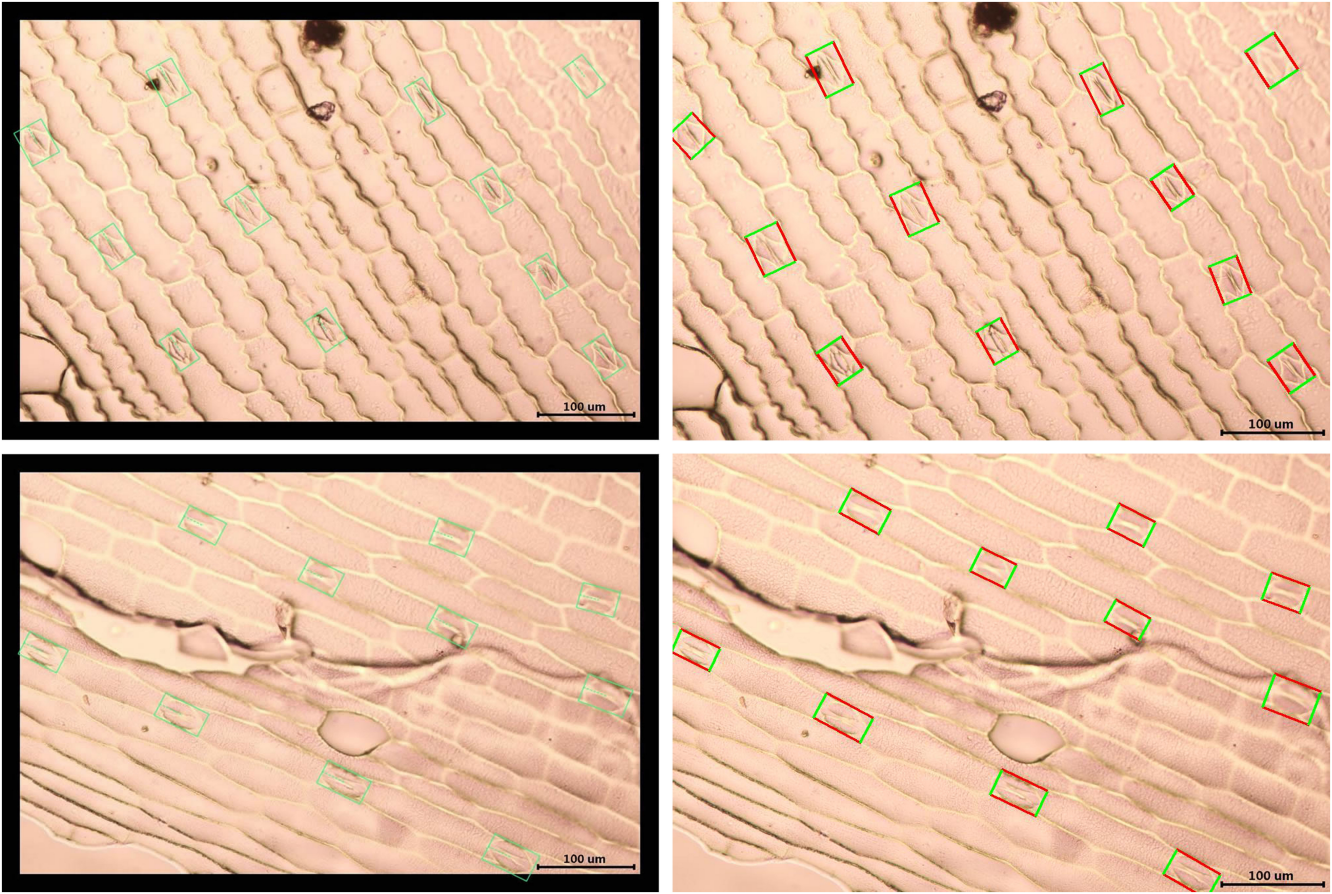
Stomata identification results. The left column images are the ground-truth images manually labeled using labelImg2 software. The right column images are the stomata images detected by our deep learning model.

Once the DL models have been trained, the detection of stomata becomes remarkably quick. Each DL model takes only approximately 0.2 s to recognize stomata within a single image. The findings of our experiments on stomata recognition are illustrated in Fig. 8. The left column images are the ground-truth images manually labeled using labelImg2 software. The right column images are the stomata images detected by our DL model. In order to label the stomata located at the image boundaries, we applied a boundary filling (adding a 30-pixel-wide black border around each image).

### Stomata segmentation and conductance calculation

Table 1 presents the results of stomata segmentation and conductance calculation, where the average length and width are in μm and the stomata conductance is in mol m^−2^ s^−1^. Our method can directly locate the stomata position in one step by rotating the enclosing box directly, which can fit the stomata better compared with the horizontal object detection method so that the parameters of stomata length and width can be measured accurately without error, and then the conductance value can be calculated by combining with the maximum stomata conductance algorithm.

**Table 1.** Stomata feature measurement results.

Images	Average length (μm)	Average width (μm)	Number	Stomata density (number mm^−2^)	Gsmax (mol m^−2^ s^−1^)
1	40.17	13.55	11	42.22	1.31
2	41.93	13.23	11	42.22	1.40
3	44.13	16.41	10	38.38	1.27
4	37.68	14.17	14	53.74	1.52
5	42.09	14.35	14	53.74	1.75
6	46.04	17.01	9	34.54	1.20
7	31.92	16.85	14	53.74	1.14
8	33.31	17.79	13	49.90	1.11
9	38.54	15.65	12	46.06	1.30
10	45.73	17.76	12	46.06	1.56
11	41.41	12.29	12	46.06	1.53
12	31.91	14.18	16	61.42	1.39

## Discussion

### Performance of the DeepRSD model

**Fig. 9.**
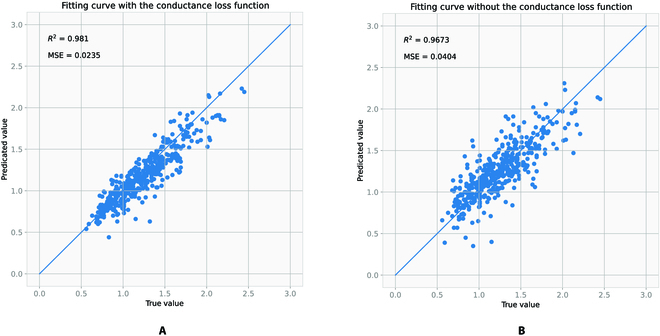
Stomata conductance value regression comparison graph. (A) Fitting result with stomata conductance loss function. (B) Fitting result without stomata conductance loss function.

Figure 9 presents a visualization of the stomata conductance measurement data and compares the results of the stomata conductance fitting. The conductance fitting results after incorporating the stomata conductance loss function are shown in Fig. 9A. Figure 9B shows the fitting result without introduction of the stomata conductance loss function. Obviously, with the addition of stomata conductance loss function, the fitting results reflect the actual measured values more accurately and the fitting accuracy is higher.

We divide the dataset into a training set, a validation set, and a test set. The training set and validation set are used for model learning training. The analyses in Fig. 9 are performed on the test set. The test set is not involved in the training process. The test set is unfamiliar data to the model so that the analysis is more convincing.

To further assess the fitting effectiveness, we computed both the MSE (mean squared error) and R-squared values. The MSE value measures the difference between the predicted value and actual values of the DeepRSD model, with a smaller value indicating a better-fitting effect. On the other hand, the R-squared value gauges how well the model fits the data, with a larger value signifying a superior fitting effect. Based on the computation results, it can be concluded that the derivative fitting result, following the addition of the derivative loss, shows a smaller MSE value and a larger R-squared value, indicating an improved fitting effect.

The main content of our experiments in this paper is the detection of maize stomata in microscopic images and the calculation of stomata maximum conductance. Our object detection method used at this stage is not able to analyze the opening and closing of stomata for the time being. The stomata opening and closing classification is our future research direction.

### Comparison with other methods

We have done a series of comparative experiments with other models, including CFA [38], ConvNeXt [39], G-Rep [40], SASM [41], KLD [42], Oriented R-CNN [43], R3Det [44], ReDet [45], Rotated FCOS [46], RetinaNet [47], and the DeepRSD model. The results were analyzed and presented in Table 2. The findings suggest that the DeepRSD model generally outperforms the other models in terms of Precision, Recall, and F1 score values for 20 epochs.

**Table 2.** The performance comparison of the different methods.

Methods	Precision	Recall	F1 score
CFA [38]	0.891	0.923	0.912
ConvNeXt [39]	0.916	0.942	0.928
G-Rep [40]	0.925	0.947	0.935
SASM [41]	0.936	0.964	0.949
KLD [42]	0.904	0.921	0.912
Oriented R-CNN [43]	0.921	0.946	0.933
R3Det [44]	0.922	0.957	0.939
ReDet [45]]	0.938	0.970	0.953
Rotated FCOS [46]	0.926	0.956	0.940
RetinaNet [47]	0.897	0.918	0.907
DeepRSD	0.943	0.974	0.958

In the realm of stomata recognition, the use of DL techniques can vastly enhance detection precision and speed, surpassing the capabilities of alternative approaches. This paper proposes a DeepRSD DL model that incorporates the features of rotating stomata to accurately determine key traits such as length and width, based on the stomata detection box size. Subsequently, the maximum stomata conductance algorithm is employed to calculate the maximum stomata conductance. The experimental results demonstrate that the DeepRSD model exhibits superior Precision, Recall, and F1 score values in comparison to other models.

However, when analyzing the detection results, we found that some labeled stomata cannot be detected by the DL model and may have been missed due to impurities in the leaves or occlusion. In addition, some substances such as small air bubbles or water droplets similar to stomata are detected by mistake. To address these problems and improve recognition accuracy, we consider improving the stomata recognition method in future experiments to further reduce false and missed detection rates.

To train a DL model effectively, a significant amount of training data is typically necessary. However, the stomata image dataset used in this paper only consists of 2,192 images, which is insufficient to support the training of a DL model. To overcome this limitation, we utilized data augmentation techniques to expand the original dataset to 24,112 images. To train the DeepRSD model, we employed the supervised learning training approach that relies on pre-classified data containing image and label information. In this study, we manually labeled each stoma. The labeling information played an essential role in training the model as a supervised signal. Due to the effectiveness of supervised learning, we were able to train a highly accurate model for stomata identification and stomata conductance calculation.

## Conclusions

This paper proposes a novel approach that utilizes DL technology to automatically recognize rotating stomata and calculate the stomata conductance of maize leaves. Our method can enhance the efficiency and accuracy of stomata recognition, thus reducing human errors. Experimental results demonstrate that the proposed anchor-free stomata detection and measurement method is both rapid and reliable. Our model identifies all stomata in a single image in just 0.2 s with high accuracy. This methodology has the potential to aid botanists in large-scale analysis of stomata traits, physiological activities, and stomata conductance, which could contribute to a better understanding of stomata responses to environmental stressors (e.g., water and soil salinization), and facilitate research on crop yield and plant stress resistance. Furthermore, this method is also applicable to other monocotyledons. This DL-based approach for automatic stomata identification and stomata conductance calculation of maize leaves not only enhances efficiency and accuracy but also offers botanists a more comprehensive and in-depth research tool.

## Data Availability

The code of anchor-free stomata detection and stomata conductance calculation has been hosted to GitHub and is available at https://github.com/sswangbo159357/Rotating-stomata-detection.
